# The product of the Herpes simplex virus 1 UL7 gene interacts with a mitochondrial protein, adenine nucleotide translocator 2

**DOI:** 10.1186/1743-422X-5-125

**Published:** 2008-10-22

**Authors:** Michiko Tanaka, Tetsutaro Sata, Yasushi Kawaguchi

**Affiliations:** 1 Department of Pathology, National Institute of Infectious Diseases, Shinjuku-ku, Tokyo 162-8640, Japan; 2 Department of Infectious Disease Control, International Research Center for Infectious Disease, The Institute of Medical Science, The University of Tokyo, Tokyo 108-8639, Japan

## Abstract

The herpes simplex virus 1 (HSV-1) UL7 gene is highly conserved among *herpesviridae*. Since the construction of recombinant HSV-1 with a mutation in the UL7 gene has not been reported, the involvement of HSV-1 UL7 in viral replication has been unclear. In this study, we succeeded in generating a UL7 null HSV-1 mutant virus, MT102, and characterized it. Our results were as follows. (i) In Vero cells, MT102 was replication-competent, but formed smaller plaques and yielded 10- to 100-fold fewer progeny than the wild-type virus, depending on the multiplicity of infection. (ii) Using mass spectrometry-based proteomics technology, we identified a cellular mitochondrial protein, adenine nucleotide translocator 2 (ANT2), as a UL7-interacting partner. (iii) When ANT2 was transiently expressed in COS-7 cells infected with HSV-1, ANT2 was specifically co-precipitated with UL7. (iv) Cell fractionation experiments with HSV-1-infected cells detected the UL7 protein in both the mitochondrial and cytosolic fractions, whereas ANT2 was detected only in the mitochondrial fraction. These results indicate the importance of HSV-1 UL7's involvement in viral replication and demonstrate that it interacts with ANT2 in infected cells. The potential biological significance of the interaction between UL7 and ANT2 is discussed.

## Introduction

Herpes simplex virus 1 (HSV-1) has a double-stranded DNA genome of about 152 kbp, from which more than 84 ORFs are translated. Since Post and Roizman first characterized recombinant viruses in which a specific HSV-1 gene was mutated by the reverse genetics system [1], this gene's roles in the viral life cycle have been extensively investigated. By now, there remain only a handful of HSV-1 genes whose roles have not been investigated using a recombinant virus with a mutated gene. The UL7 gene, the subject of this study, is one such viral gene. The UL7 amino acid sequence is conserved in all *Herpesviridae *subfamilies [2], suggesting that UL7 homologues may play conserved roles in the herpes virus life cycle. The viral gene is on the left side of the HSV-1 unique long (U_L_) region and surrounded by two essential viral genes (UL6 and UL8) for virus replication in cell cultures [3]. The UL7 gene partially overlaps with the UL6 gene, and these transcripts are coterminal at their 3' ends. Information on the function(s) of the HSV UL7 gene product in the viral life cycle is limited. The only reported experimental evidence with regard to HSV UL7 is that its gene products are present in integumentary layers of mature virions, and that the viral protein is localized predominantly in the juxtanuclear cytoplasmic domains of infected cells, although it is also detected transiently in the nucleus [4]. On the other hand, mutant viruses in which the UL7 homologous genes of other alphaherpesviruses pseudorabies virus (PRV) and bovine herpesvirus 1 (BHV-1) have been constructed and characterized [5,6]. The mutant viruses revealed that the UL7 homologous genes are dispensable for viral replications of PRV and BHV-1, although the mutant viruses exhibit impaired capacity to replicate in cell cultures. These results indicate that the UL7 homologous genes of PRV and BHV-1 are involved in viral replication in cell cultures. However, the mechanisms underlying the actions of the gene products in viral replication are unclear. In the present study, we succeeded in generating a UL7 null mutant virus and characterizing it in cell cultures. Furthermore, as a first step to elucidating the mechanism by which UL7 functions in viral replication, we attempted to identify cellular proteins that interact with UL7.

## Materials and methods

### Cells and viruses

Vero, rabbit skin, and COS-7 cells were maintained in Dulbecco's modified Eagle's medium (DMEM) containing 5% fetal calf serum (FCS) as described previously [7]. 293T cells were maintained in DMEM containing 10% FCS. The recombinant virus YK304 was reconstituted from pYEbac102, which contained a complete HSV-1(F) sequence with the bacterial artificial chromosome (BAC) sequence inserted into the HSV intergenic region between UL3 and UL4 [8]. YK304's phenotype has been shown to be identical that of the wild-type HSV-1(F) in cell cultures and in mouse models [8].

### Plasmids

pcDNA-MEF [9], in which the myc-TEV-Flag (MEF) tag cassette was inserted into the multi-cloning sites in the mammalian expression vector pcDNA3 (Invitrogen), was kindly provided by Dr. T. Suzuki. To construct pMEF7, a UL7 expression vector whose UL7 gene is tagged with both Flag and Myc epitope sequences, a UL7 open reading frame (ORF) without a start codon was amplified by polymerase chain reaction (PCR) from the HSV-1 genome and inserted into the *Eco*RI and *Xba*I sites of pcDNA-MEF. To construct expression vector pCMV(f)7, whose UL7 gene is tagged with only the Flag epitope sequence, a UL7 ORF without a start codon was PCR amplified and inserted into the *Eco*RI and *Bam*HI sites of pFLAG-CMV-2 (Sigma). To construct pTeasy-ANT, the ANT2 ORF was PCR amplified from a human cDNA library (kindly provided from Dr. Y. Kawaguchi) and cloned into pGEM-T Easy (Promega). pCMV(f)ANT was constructed by amplifying the ANT2 ORF from pTeasy-ANT and cloning it into the *Eco*RV and *Xba*I sites of pFLAG-CMV-2. pBS-XH2.2 was constructed by cloning a 2.2 kbp fragment containing a UL7 ORF amplified from the HSV-1 genome by PCR into pBluescript II KS+ (Stratagene).

### Mutagenesis of viral genomes in E. coli and generation of recombinant viruses

First, we generated a UL7 mutant virus genome (pMT101) in which a domain of UL7 encoding codons 27–891 was replaced with the gene encoding kanamycin resistance using a one-step mutagenenesis method called ET cloning as described previously [10]. Briefly, linear fragments containing a kanamycin-resistant gene, FRT sequence, and 50 bp flanking of UL7 sequences on each side, were generated by PCR from pCR2.1 (Invitrogen) using the following primers: 

5'-AGGGCGGGGGCATCGGGCACCGGGATGGCCGCCGCGACGGCCGACGATG

AGAAGTTCCTATTCTCTAGAAAGTATAGGAACTTCGACAGCAAGCGAACCGGAAT-3' 

and 5'-CGCATCCGTCGGGAGGCCACAGAAACAAAACCGGGTTTATTTCCTAAAAT

GAAGTTCCTATACTTTCTAGAGAATAGGAACTTCCGGAAATGTTGAATACTCA

TACTCTTCCTTTTTC-3'. The linear PCR-generated fragments were electroporated into YEbac102, an *E. coli *DH10B strain containing HSV-1(F)-BAC plasmid pYEbac102 [8] and pGETrec encoding recombinases E and T (a generous gift from Dr. P. A. Ioannou) [10]. Kanamycin-resistant colonies were then screened by PCR with appropriate primers, which led to the identification of *E. coli *harboring the mutant HSV-BAC plasmid pMT101. The next step was to remove the gene encoding kanamycin resistance from pMT101. To this end, the Flp expression plasmid pCP20Zeo [11] was electroporated into the *E. coli *harboring pMT101 as described previously [8]. Kanamycin-sensitive colonies were screened by PCR with appropriate primers to confirm the loss of the kanamycin resistance gene, which led to the identification of *E. coli *harboring pMT102. The UL7 deletetion mutant virus MT102 was generated by the transfection of rabbit skin cells with pMT101. In the recombinant virus MT103, the original UL7 sequence in MT102 was restored by cotransfecting MT102 DNA with pBS-XH2.2. Plaques were isolated and screened for the presence of a UL7 sequence. The recombinant viruses were verified by Southern blotting as described previously [12].

### Antibodies

Rabbit polyclonal antibodies to UL7 and UL49 [13] were kindly provided by Dr. Y. Nishiyama. Mouse monoclonal antibody to Flag epitope (M2) and mouse monoclonal antibody to βactin were purchased from sigma and mouse monoclonal antibody to COX IV was purchased from Invitrogen.

### Quantitative RT-PCR

Relative quantification of UL6 and UL8 to 18S rRNA was performed in a Thermal Cycler Dice Real Time System (Takara) by real-time RT PCR. Total RNA was extracted from Vero cells infected with YK304, MT102, or MT103 at an MOI (multiplicity of infection) of 5 for 20 h, and residual DNA was digested with DNase I by the SV Total RNA Isolation System (Promega). cDNA was synthesized using the PrimeScript RT-PCR reagent kit (Takara) according to the manufacturer's instructions. Real-time PCR amplifications were performed with primers UL6-f (5'-aaattctgtgtcaccgcaacaac-3') and UL6-r (5'-gcccgaagcactgactcaa-3') for UL6; UL8-f (5'-cttgctggacgcagagcacta-3') and UL8-r (5'-gatttcgcgcaggtgatgag-3') for UL8; and 18S rRNA-f (5'-actcaacacgggaaacctca-3') and 18S rRNA-r (5'-aaccagacaaatcgctccac-3') for 18S rRNA. Reactions were performed using SYBER Premix Ex Taq II (Takara) with the Thermal Cycler Dice Real Time System. Template-negative and RT-negative reactions served as controls.

### MEF purification

MEF purification was performed as described previously [9] with minor modification. Briefly, 293T cells in 10 100-mm dishes were transfected with 6 μg of pcDNA-MEF or pMEF7 per dish using FuGENE 6 (Roche Applied Science). At 48 h post-transfection, cells were harvested, washed with phosphate-buffered saline (PBS), and lysed in 5 ml of NP40 buffer (50 mM Tris-HCl (pH 8.0), 120 mM Nacl, 0.5% NP40, and 1 mM phenylmethylsulfonyl fluoride (PMSF)). The supernatants obtained after centrifugation were passed through filters with a pore size of 0.22 μm and precleared by mixing with protein G-Sepharose beads for 30 min at 4°C. The supernatants obtained after centrifugation and filtration were reacted with 100 μl of Sepharose-conjugated anti-myc antibody (MBL) for the first immunoprecipitation. After incubation for 90 min at 4°C, the beads were washed four times with NP40 buffer and once with TEV buffer (Invitrogen). The beads were then reacted with 10 units of AcTEV protease (Invitrogen) in 100 μl of TEV buffer containing 0.1 M DTT at room temperature for 60 min with rotation to release bound materials from the beads. After the supernatants were collected by centrifugation, the beads were washed twice with TEV buffer (70 μl). The resultant supernatants were combined and reacted with 1 μl of anti-Flag monoclonal antibody (M2) for 2 h at 4°C for a second immunoprecipitation. Then, 30 μl of protein G-Sepharose beads was added and allowed to react for an additional 1 h at 4°C. The beads were then washed three times with NP40 buffer and subjected to electrophoresis in a denaturing gel. The immunoprecipitates were visualized by silver staining (Daiichikagaku, Japan) according to the manufacturer's instructions. They were excised and digested in the gel with trypsin, then analyzed by a mass spectrometer, MALDI-TOF MS (Voyager-DE STR; Applied Biosystems).

### Coimmunoprecipitation and immunoblotting

Coimmunoprecipitaion and immunoblotting were performed as described previously [14]. Briefly, COS-7 cells in 60-mm dishes were transfected with pCMV(f)ANT in combination with pFLAG-CMV-2 or pCMV(f)7 using FuGENE 6. At 48 h post-transfection, cells were harvested, washed with PBS, and lysed in 500 μl of NP40 buffer (50 mM Tris-HCl (pH 8.0), 120 mM Nacl, 0.5% NP40, 1 mM PMSF). The supernatants obtained after centrifugation were precleared by incubation with protein G-Sepharose beads for 30 min at 4°C (GE Healthcare). After a brief centrifugation, the supernatants were reacted with the anti-UL7 rabbit polyclonal antibody for 2 h at 4°C. Protein G-sepharose beads were then added and allowed to react with rotation for an additional 1 h at 4°C. The immunoprecipitates were collected by a brief centrifugation, washed extensively with NP40 buffer, and analyzed by immunoblotting with anti-Flag monoclonal antibody. In other experiments, COS-7 cells were transfected with pFLAG-CMV-2 or pCMV(f)ANT as described above. At 24 h post-transfection, transfected cells were infected with YK304 or MT102 at an MOI of 5. At 24 h after infection, the cells were harvested and subjected to immunoprecipitation with the UL7 antibody and immunoblotting with the anti-Flag antibody as described above.

### Subcellular fractionation

Subcellular fractionation was performed as described previously [15]. Briefly, COS-7 cells in 100-mm dishes were transfected with pCMV(f)ANT as described above. At 24 h post-transfection, cells were mock-infected or infected with YK304, MT102, or MT103 at an MOI of 5. At 24 h after infection, cells were harvested and resuspended in 0.8 ml of ice-cold buffer A (20 mM HEPES, 10 mM KCl, 1.5 mM MgCl2, 1 mM EDTA, 1 mM EGTA, 1 mM dithiothreitol, 250 mM sucrose) containing a protease inhibitor cocktail. After incubation for 15 min on ice, the samples were homogenized in a Dounce homogenizer and then centrifuged for 10 min at 750 g. The supernatants were transferred to new tubes and centrifuged again at 10,000 g per 20 min. Supernatants from the second centrifugation were concentrated by acetone precipitation and represented the cytosolic fraction, whereas the pellets represented the mitochondrial fraction.

## Results

### Generation of a UL7 deletion mutant virus and its repaired virus

**Figure 1:**
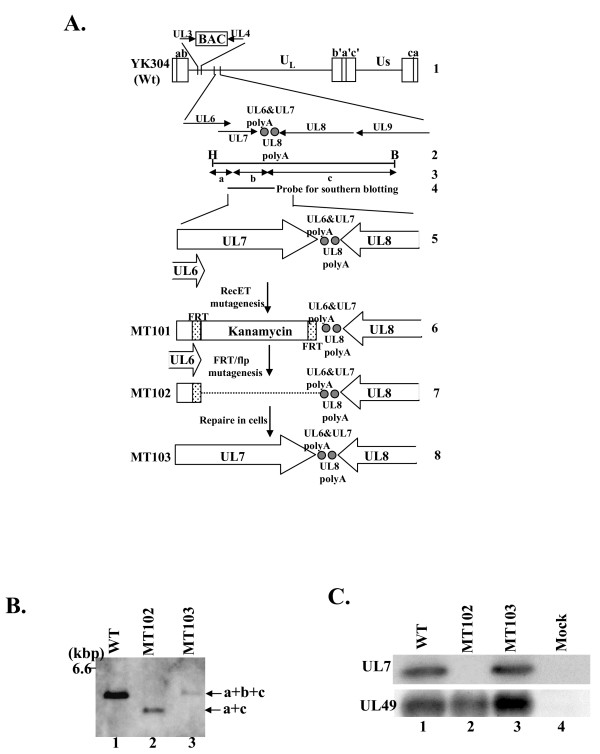
**Strategy and construction of the recombinant virus MT101, 102, and 103**. (A) Schematic diagram of genome structures of wild-type YK304 and relevant domains of the recombinant viruses. Line 1, a linear representation of the YK304 genome. The YK304 genome has bacmid (BAC) in the intergenic region between UL3 and UL4. Line 2, the genomic domain encoding UL6 to UL9 open reading frames. The DNA fragment and restriction enzyme sites in the genomic domain encoding UL6 to UL9 open reading frames. Line 3, expected sizes of DNA fragments generated by cleavage of DNA. The fragment designations shown here are identical to those described in the text and in Fig. 1B. Line 4, location of the DNA fragment used as a radiolabeled probe in Fig. 1B. Line 5, an expanded section of the parts of UL6, UL8 and whole of UL7 open reading flame. Line 6, a schematic diagram of the recombinant virus genome. As a result of RecET mutagenesis, a kanamycin-resistant cassette was inserted into a truncated UL7 gene that contained an HSV-BAC maintained in an *E. coli*. Line 7, a schematic diagram of the recombinant virus MT102. As a result of flp-mediated site-specific recombination, the kanamycin-resistant gene was excised from the virus genome and a single FRT site remained. MT102 was reconstituted by transfection of the mutated HSV-BAC (pMT102) into rabbit skin cells. Line 8, a schematic diagram of the repaired virus MT103. The rescue of MT102 by cotransfection of its DNA was the same as that used for the radiolabeled probe. Restriction sites: H, *Hind*III; B, *Bam*HI. (B) Autoradiographic images of electrophoretically separated *Bam*HI and *Hind*III digests of YK304 (lane 1), MT102 (lane 2), and MT103 (lane 3) DNAs hybridized to the radiolabeled DNA fragment of HSV(F) described in line 4 of Figure 1A. The letters on the right refer to the digests of the DNA fragments generated by restriction endonuclease cleavage. (C) Photographic image of the immunoblots of electrophoretically separated lysates of Vero cells infected with wild-type YK304 (lane 1), MT102 (lane 2), or MT103 (lane 3). The infected cells were harvested at 18 h post-infection and subjected to immunoblotting with the rabbit polyclonal antibody to UL7 (upper panel). The same membrane was re-labeled with the rabbit polyclonal antibody to UL49 (lower panel).

To explore the necessity of UL7 during HSV-1 infection in cultured cells, UL7 deletion mutant virus MT102 and its repaired virus MT103 were generated. The strategy for constructing the recombinant viruses is summarized in Figure 1A. The UL7 deletion mutant virus was able to be reconstituted by transfection of pMT102, which contains a deletion in the UL7 locus of the HSV-1 genome, into rabbit skin cells. This reconstitution indicated that UL7 is not essential for HSV-1 replication in cell culture. To verify the genome structures of the recombinant viruses, each viral genome extracted from cells infected with YJ304, MT102, or MT103 was digested with BamHI and HindIII, electrophoretically separated, and analyzed by Southern blotting with a DNA fragment probe as shown in Figure 1A, line 4. As expected, the probe hybridized to fragment a + b + c (6.0 kbp) in YK304 and repaired virus MT103 (Figure 1B, lanes 1 and 3) and fragment a + c (5.4 kbp) in UL7 deletion mutant MT102 (Figure 1B, lane 2). UL7 protein expression was examined by immunoblotting. Vero cells mock-infected or infected with YK304, MT102, or MT103 were harvested 20 h after infection and were analyzed by using anti-UL7 antibody. As expected, UL7 protein was not detected in mock- or MT102-infected cell lysates (Figure 1C), whereas UL49 protein levels were equivalent among all of the lysates of infected cells.

Although we engineered our UL7 mutant virus to avoid disrupting expression at neighboring loci, the UL6 gene overlaps with the UL7 gene and the UL8 gene is only 3' to the UL7 gene. Therefore, we next examined whether or not deletion of the UL7 sequence influences expression from neighboring loci, using real-time RT-PCR to quantitate the expression of UL6 and UL8 genes in Vero cells infected with YK304, MT102, and MT103 at 20 h after infection. The results were that the expression levels of UL6 and UL8 genes in Vero cells infected with MT102 (ΔUL7) were similar to those in Vero cells infected with wild-type YK304 and MT103 (repair) (data not shown). These results indicate that deletion of the UL7 sequence from the HSV-1 genome has no effect on the expression of neighboring genes.

### Growth properties of the UL7 deletion mutant virus in Vero cells

**Figure 2:**
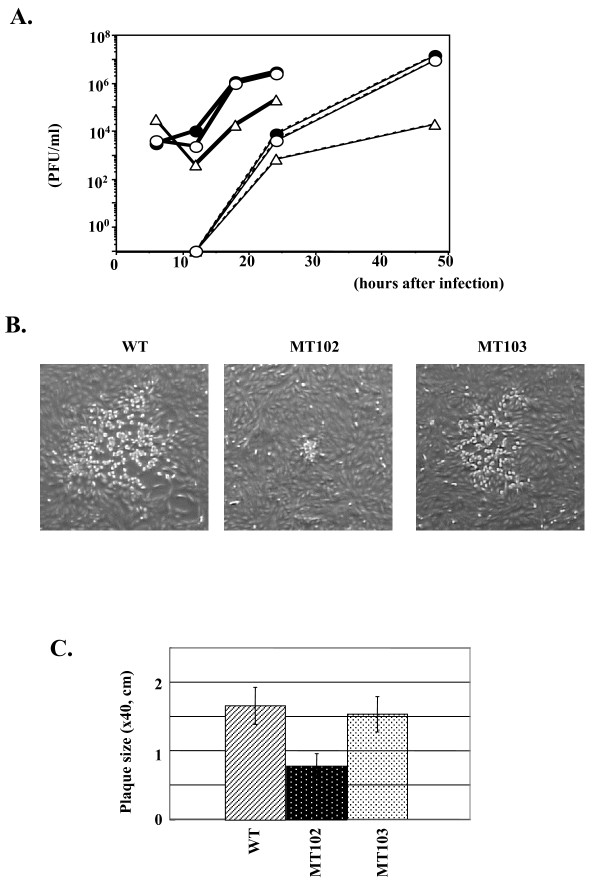
**Comparison of the phenotype of wild-type YK304 and the recombinant viruses MT102 and MT103**. (A) Vero cells were infected with YK304 (filled circles), MT102 (open triangles), or MT103 (open circles) at a multiplicity of 3 (thick lines) or 0.01 (thin lines) PFU per cell. The supernatants and cells were harvested at the indicated time points, and cell lysates were titrated on Vero cells. (B) Photographs of plaque produced by wild-type YK304 (left panel), MT102 (middle panel), and MT103 (right panel). Vero cells infected with each of the recombinant viruses at an MOI of 0.0001 PFU per cell under plaque assay conditions. Phase-contrast photographs were recorded 2 days after infection. (C) The mean diameters of 20 single plaques per recombinant virus were determined.

To examine the role of the UL7 gene product in viral growth in cell cultures, two series of experiments were performed. First, Vero cells were infected with wild-type YK304, MT102 (ΔUL7), or MT103 (repair) at an MOI of either 3 or 0.01; the total virus yield from the cell culture supernatants and the infected cells were harvested at the indicated time points (Figure 2A). The titers of each sample were determined by standard plaque assays on Vero cells. As shown in Figure 2A, the ability of the UL7 deletion mutant MT102 to replicate in Vero cells is apparently impaired. Multi-step growth analysis (MOI = 0.01) indicated that the viral titer of MT102 (ΔUL7) was reduced nearly 100-fold compared to that of wild-type YK304 at 48 h post-infection. Even at an MOI of 3, the yield of MT102 (ΔUL7) at 24 h post-infection was about 10-fold less than that of wild-type YK304 (Figure 2A). The growth curves of MT103 (repair) at MoI of 0.01 and 3 were almost the same as those of parental virus YK304, indicating that the growth defect observed in MT102 (ΔUL7) was indeed due to the loss of the UL7 sequence. Similar results were obtained in repeated experiments (data not shown).

In the second series of experiments, Vero cells were infected with wild-type YK304, MT102 (ΔUL7), or MT103 (repair) under the conditions for plaque assay, and plaque sizes were analyzed 2 days after infection. As shown in Figure 2B, MT102 produced remarkably smaller plaques (middle panel) than both wild-type YK304 and MT103 (repair) (left and right panels). The differences in plaque size were statistically significant (Figure 2C; P < 0.001). Similar results were obtained in repeated experiments (data not shown). These results indicate that UL7 is necessary for the efficient replication of HSV-1 in cultured cells.

### Identification of ANT2 as a UL7-interacting protein

**Figure 3:**
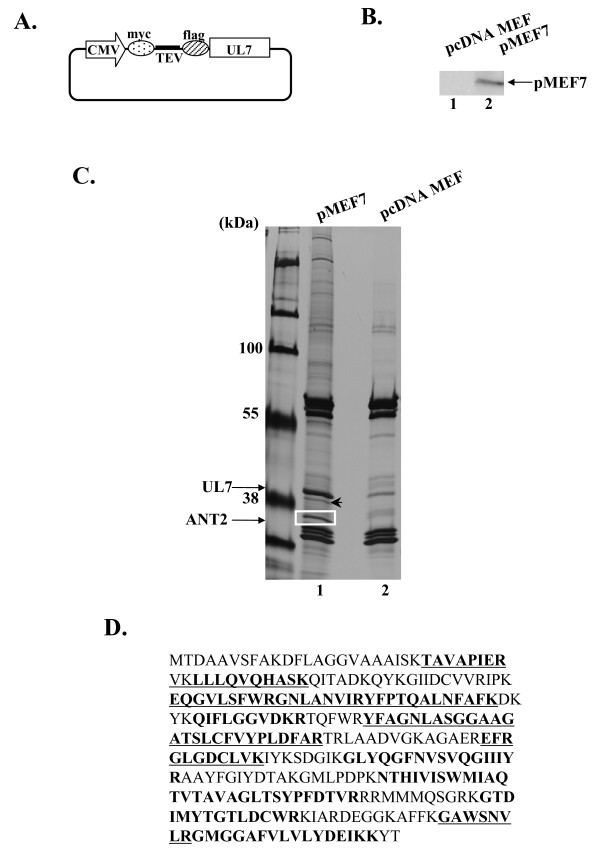
**Identified host proteins interact with UL7 by using MEF purification method**. (A) Schematic diagrams of the expression plasmid containing UL7 tagged with the myc, TEV protease, and flag. (B) Photograph of an immunoblot of electrophoretically separated lysates of COS-7 cells transfected with pcDNA MEF (lane 1) or pMEF7 (lane 2) and subjected to immunoblotting with the mouse monoclonal antibody to the flag epitope. (C) Photograph of electrophoretically separated lysates of 293T cells. 293T cells transfected with pMEF (lane 1) or pcDNA MEF (lane 2) were harvested, lysed, and immunoprecipitated as described in Materials and Methods. The proteins bound to UL7 were purified and separated with 10% SDS-page and silver-stained (lane 1). The bands surrounded by the white rectangle and indicated by the arrowhead were subjected to a mass spectrometry experiment. (D) Peptide sequence of ANT2. The sequences detected by mass spectrometry and specific for ANT2 are shown in bold type. The sequences conserved in ANT 1~3 or ANT1~4 are shown in bold type and underlined.

In our first step in attempting to clarify the function(s) of UL7 in viral replication, we tried to identify the host cellular proteins that interact with the UL7 protein. To identify such proteins, we adopted the tandem affinity purification approach coupled with mass spectrometry-based proteomics technology [9]. To purify cellular proteins that interact with the UL7 protein, we employed original N-terminal affinity tags, myc and Flag, that were fused in tandem and separately by a spacer sequence containing a TEV protease cleavage site (myc-TEV-Flag) (Figure 3A). UL7 protein tagged with MEF was purified with its binding proteins from the lysates of 293T cells in which the MEF-UL7 protein was transiently expressed (Figure 3B), and the UL7 binding proteins were identified by mass spectrometry. Figure 3C shows profiles of the immunoprecipitates containing MEF-UL7 and its binding proteins in a denaturing gel. Several bands that were detected in immunoprecipitates of the lysates of cells transfected with pMEF7, but not with the empty vector pcDNA-MEF, were excised and subjected to gel digestion and mass spectrometry analysis. The protein in the band surrounded by the white box in Figure 3C was identified as ANT2, which was located on the inner mitochondrial membrane. Another protein, indicated by the arrowhead, was ANT4 (SLC25A3), which was also an inner mitochondrial membrane protein.

### UL7 interacts with ANT2 in mammalian cells and in HSV-1-infected cells

**Figure 4:**
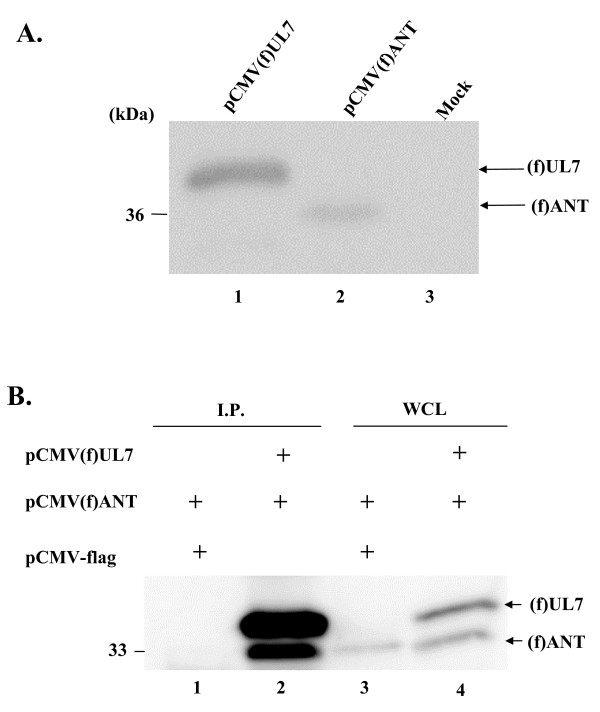
**Interaction between UL7 and ANT2 in mammalian cells**. (A) Photograph of an immunoblot of electrophoretically separated lysates of COS-7 cells transfected with pCMV(f)UL7 (lane 1) or pCMV(f)ANT (lane 2) and subjected to immunoblotting with the antibody to the flag epitope. (B) COS-7 cells transfected with the indicated expression plasmids were immunoprecipitated with antibody to the UL7. The immunoprecipitates were subjected to electrophoresis on a denaturing gel, transferred to a PVDF sheet, and reacted with the flag antibody. Four percent of the COS-7 whole cell extracts (WCE) input to the immunoprecipitation reactions for lanes 1 and 2 were loaded into lanes 3 and 4, respectively.

To verify whether or not UL7 in fact associates with ANT2 in cultured cells, pCMV(f)UL7 and pCMV(f)ANT expressing Flag epitope-tagged UL7 and ANT2, respectively, were constructed. The expression level of each protein tagged with Flag epitope in transfected cells was verified by immunoblotting with anti-Flag antibody (Figure 4A). COS-7 cells transfected with the indicated expression vectors (Figure 4B) were solubilized and immunoprecipitated with the anti-UL7 polyclonal antibody. The immunoprecipitates were then subjected to immunoblotting with the anti-Flag antibody. As shown in Figure 4B, the UL7 antibody coprecipitated UL7 with Flag epitope-tagged ANT2 when UL7 and ANT2 were coexpressed in COS-7 cells (lane 2). In contrast, when ANT2 was expressed by itself, the antibody did not precipitate ANT2 (lane 1). Immunoblotting of whole cell extract from transfected cells indicated that each protein tagged with Flag epitope was appropriately expressed in COS-7 cells. These observations indicate that UL7 interacts with ANT2 in mammalian cells.

**Figure 5:**
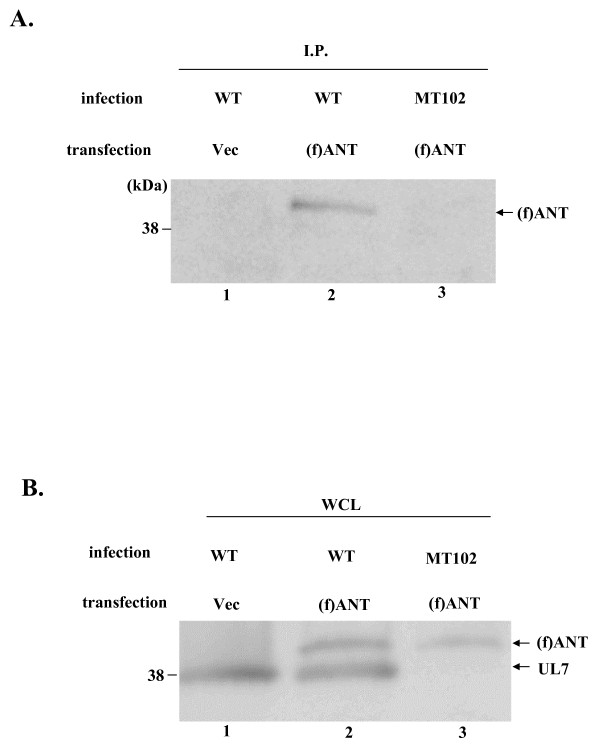
**Interaction between UL7 and ANT2 in super-infected cells**. (A) COS-7 cells infected with the indicated virus that transiently expressed (f)ANT2 were immunoprecipitated with rabbit polyclonal antibody to UL7. The immunoprecipitates were subjected to electrophoresis on a denaturing gel, transferred to a PVDF sheet, and reacted with the flag antibody. (B) Four percent of the COS-7 WCE input to immunoprecipitation reactions for lanes 1, 2, and 3 were loaded into lanes 1, 2, and 3, respectively.

Next, COS-7 cells were transfected with pCMV(f)ANT (Figure 5A and 5B, lanes 2 and 3) or pFlag-CMV2 (Figure 5A and 5B, lane 1). At 24 h after transfection, the transfected cells were infected with wild-type YK304 or MT102 (ΔUL7) at an MOI of 5. At 24 h post-infection, infected cells were harvested, solubilized, and immunoprecipitated with the anti-UL7 antibody. The immunoprecipitates were then subjected to immunoblotting with anti-Flag antibody. As shown in Figure 5, the anti-UL7 antibody coprecipitated with Flag epitope-tagged ANT2 from cells infected with wild-type YK304 (lane 2), while it did not do so from cells infected with MT102 (ΔUL7) (lane 3). Immunoblotting of whole cell extract indicated that ANT2 tagged with Flag epitope and UL7 were appropriately expressed in COS-7 cells. These results indicate that UL7 interacts with ANT2 in HSV-1 infected cells.

### HSV-1 UL7 was detected in the mitochondrial fraction of HSV-1-infected cells

**Figure 6:**
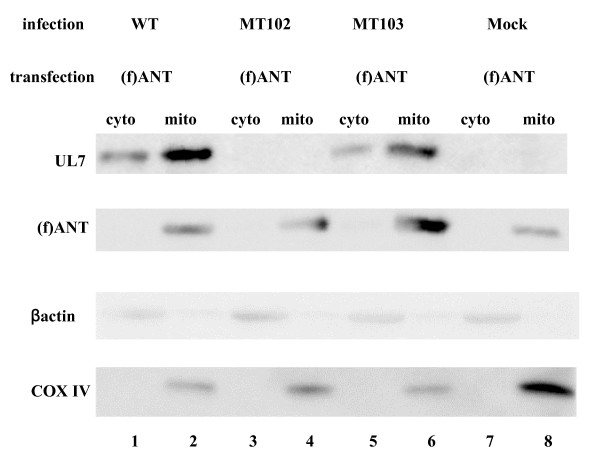
**Both of UL7 and ANT2 were exist in the mitochondrial fractions**. Photograph of electrophoretically separated lysates of COS-7 cells infected with the indicated virus that transiently expressed (f)ANT2. The cytosolic fractions (lanes 1, 3, 5, and 7) and mitochondrial fractions (lanes 2, 4, 6, and 8), separated as described in Materials and Methods, were subjected to electrophoresis on a denaturing gel, transferred to a PVDF sheet, and reacted with the antibody to UL7 (first panel). The same membrane was reacted with the antibosy to the flag again (second panel), to the βactin (third panel) and to COX IV (bottom panel).

ANT proteins localized specifically in the inner mitochondrial membrane. The result that UL7 interacts with ANT2 in infected cells suggests that UL7 is a mitochondrial viral protein in infected cells. To test this hypothesis, COS-7 cells were transfected with pCMV(f)ANT and then, at 24 h after transfection, the transfected cells were mock-infected or infected with wild-type YK304 or MT102 (ΔUL7) at an MOI of 5. At 24 h post-infection, infected cells were harvested and subjected to cell fractionation experiments, and each fraction was subjected to immunoblotting with the anti-UL7 and anti-Flag antibodies. As shown in Figure 6, Flag-tagged ANT-2 and COX IV, also one of mitochondrial membrane protein were specifically detected in the mitochondrial fraction of mock-infected and infected Vero cells and βactin was specifically detected in the cytosolic fraction, suggesting that cell fractionation was appropriately performed. UL7 proteins accumulated mainly in the mitochondrial fraction of COS-7 cells infected with wild-type YK304, although the proteins also accumulated in the cytosolic fraction. These results suggest that UL7 is in fact a mitochondrial protein in infected cells.

## Discussion

The essentiality of HSV-1 UL7 in viral replication in cell cultures has been controversial (Roizman & Knipe, 2001), and no experimental evidence supporting the assumptions of essentiality has been reported. In the present study, we have constructed a null mutant virus of HSV-1 UL7, called MT102, and presented evidence that MT102 is able to replicate in Vero cells, indicating that the HSV-1 UL7 gene is dispensable in HSV-1 replication in cell culture. Interestingly, both the plaque-forming ability and the viral growth of MT102 in cell culture were greatly impaired compared to those of the wild-type virus. This impairment of MT102's growth properties is due solely to the deletion of the UL7 gene, for two reasons: first, UL7 gene deletion did not affect the expression of neighboring genes UL6 and UL8, both of which are essential for viral replication in cell culture; and second, the repair of the UL7 gene deletion (MT103) restored the wild-type growth properties. These phenotypes of the UL7 null mutant virus of HSV-1 are consistent with those of PRV and BHV-1. Taken together, these observations indicate that UL7 is significantly involved in viral replication in cell culture.

In a previous report, electron microscopic analyses of the PRV UL7 null mutant virus demonstrated that the absence of PRV UL7 did not affect the intranuclear steps of virion formation, including capsid assembly, encapsidation of viral DNA, nuclear egress of capsids, and secondary envelopment in cytoplasmic membrane vesicles, but did affect the release of finally enveloped virions from cells [5]. Consistently, the release defects of viruses have been observed with UL7 deletion mutant viruses of HSV-1 (this study) and BHV-1 [6]. These results suggest that one of the conserved roles of UL7 homologues in viral replication is to regulate virion release from infected cells. On the other hand, virus titers in cells infected with MT102 were also impaired, as observed with the UL7 null mutant viruses of BHV-1 [6] and PRV [5], implying that each UL7 protein functions in at least one step of viral replication other than viral release. Thus, UL7 homologues seem to play multiple roles in viral replication. However, the mechanism or mechanisms by which the UL7 gene product acts in infected cells remain unknown.

As a first step to elucidate such mechanisms, we attempted to identify cellular protein interacting with HSV-1 UL7 by using the MS-based proteomics technology combined with a tandem affinity purification tag, called MEF [9], and we identified ANT2 as a UL7-interacting partner. ANT is located in the inner mitochondrial membrane as a member of the permeability transposition pore (PT) complex, which comprises ANT, voltage-dependent anion channel (VDAC), hexokinase, and cyclophilin D, and regulates its functions so that they interact with each other [3,16]. ANT is a bifunctional protein that, in physiological conditions, exchanges ATP and ADP on the inner mitochondrial membrane, whereas in apoptotic conditions it can form a nonspecific pore [3,17]. Recently, ANT was reported to be a component of the mitochondrial permeability-transition pore (mtPTP); on the other hand, it is also essential for maintaining the cell metabolism exchange of cytosolic ADP for mitochondrial ATP [16]. In the present study, we demonstrated that ANT2 from COS-7 cells transfected with the ANT expression vector and infected with wild-type HSV-1 was co-precipitated with UL7. Furthermore, UL7 is detected in both the mitochondrial and cytosolic fractions in infected cells in cell fractionation experiments, which reinforced the interaction between UL7 and the mitochondrial protein. Together, these series of observations indicate that UL7 interacts with ANT2 in HSV-1-infected cells.

The biological significance of the interaction between UL7 and ANT2 is uncertain. Four ANT proteins exist in human (ANT1~4) as the mitochondrial carrier family, and they are expressed in tissue- and development-specific manners [18-20]. ANT2 is up-regulated in proliferative cells, including several cancer cell lines, and induces apoptosis by interacting with many kinds of materials [21], including viral protein (Vpr of HIV-1 and pBI-F2 of influenza virus) [22-24], although ANT2 was not an essential member. In addition, ANT2 repression results in the growth arrest of human cells; that is to say, only ANT2 negatively regulates apoptosis, and thus may be oncoprotein, despite the close similarity among the four ANT genes. ANT2 has therefore recently become a useful target for cancer therapy based on molecular targeting [25]. These reports suggest the special involvement of ANT2 in conditions of stress, not only in cancer cells but also in viral infection. In addition, some mitochondrial changes in HSV-infected cells have been reported [26,27]. Spherical morphological change of mitochondria was observed using intensified fluorescence digital imaging at an early point in infection [28]. A confocal microscopic study also reported clustering of mitochondria in HSV-2 infected cells [29]. Oxidative stress of mitochondria and Ca+ release were observed by NF-kB activation induced by HSV infection [30]. Other studies using HSV mutants, revealed the release of cytochrome C, which is known to be a stress-responsive mitochondrial protein, into the cytoplasm [31,32]; it thus confirms the influence of HSV infection on mitochondrial condition. From these facts, it is undeniable that UL7 may be involved in the control of mitochondrial functions and/or conditions through ANT2, because ANT2 is an important member of the mitochondrial inner membrane proteins that modulate mitochondrial life. It is also interesting that another ANT family member, ANT4 (SLC25A3), recently identified but with an unknown function [19], also interacts with UL7 in human cells. Finally, UL7 may modulate the functions of ANT2 or some other ANT members and rescue HSV infected cells so that they can survive the virus on their own.

## Competing interests

The authors declare that they have no competing interests.

## Authors' contributions

MT conceived this study, designed and performed the experiments and drafted the manuscript and writing. TS participated in the design of this study. YK participated in the design, coordination of this study. All authors read and approved the final manuscript.
